# RACIAL DIFFERENCES IN CONTEMPLATIVE PRACTICES IN LATER LIFE: EXAMINING THE ROLE OF SES AND HEALTH DISADVANTAGE

**DOI:** 10.1093/geroni/igz038.537

**Published:** 2019-11-08

**Authors:** Nirmala Lekhak, Tirth Bhatta, Timothy Goler

**Affiliations:** 1 University of Nevada Las Vegas, Las Vegas, Nevada, United States; 2 UNLV, Las Vegas, Nevada, United States; 3 Case Western Reserve University, Cleveland, Ohio, United States

## Abstract

Substantial scholarly attention has been placed on prayer as a buffer of life events’ adverse influences on well-being in later life. The disproportionate distribution of adverse life events among Black adults has also attracted scholarly interest in racial differences in contemplative practices. Black adults have been found to more likely engage in private prayer than White adults, whereas studies have observed an opposite pattern for meditation. The contribution of stratification in socioeconomic status and health to racial differences in contemplative practices, especially in meditation has received relatively less attention. Drawing from a subsample from Health and Retirement Survey (N = 1102), this study takes a next necessary step to assess the contribution of socioeconomic status, multimorbidity, and depressive symptoms to racial differences in both prayer and meditation use in later life. Consistent with prior studies, the odds of engaging in private prayer (OR=2.78, p<0.01) was higher among Blacks than White older adults. Our findings of higher odds (OR=2.92, p<0.001) of meditation among Black older adults than White older adults, however, do not align with previous studies. The disadvantage in socioeconomic status, health, and psychological well-being completely explain racial differences in prayer, but this difference in meditation persist even after adjusting for those factors. Our findings call for further research on contextually influenced underlying individual motivations that drive older adults of different racial and social economic groups to engage in various contemplative practices. Further research is also warranted on how older adults, particularly Blacks differentiate between private prayer and meditation.

